# Supplementary material for: Global history, the emergence of chaos and inducing sustainability in networks of socio-ecological systems
Source: PLoS One. 2023 Nov 16;18(11):e0293391. doi: 10.1371/journal.pone.0293391 (PMC10653456; doi:10.1371/journal.pone.0293391)
Supplement: S1 Text — In the appendix we propose an economic interpretation for model (1). Sunk costs effects [19] are quantified and compared with the definition of rationality in economics as utility maximization. (PDF) [file pone.0293391.s001.pdf]

Appendix A: Economic interpretation

We interpret the wealth variable z as an utility function $U(x)$ (or net benefit) of the population x (consider the "produced" quantity). By the chain rule we have that:

$$\frac{d}{dt}[U(x)] = U'(x)\dot{x} \tag{1}$$

where $U'(x)$ is marginal utility (utility gained for each unit produced, or the derivative with respect to x). Because $U(x) = z$, we have that:

$$\frac{d}{dt}[U(x)] = \dot{z} \implies U'(x) = \frac{dz}{dx} = \frac{\dot{z}}{\dot{x}} \tag{2}$$

The two contributing terms to $\dot{z} = \alpha x^2 y - cz$ can be interpreted as the time derivatives of benefit $B(x)$ and cost $C(x)$ functions:

$$\begin{aligned} \frac{d}{dt}[B(x)] &= \alpha x^2 y \\ \frac{d}{dt}[C(x)] &= cz \end{aligned} \tag{3}$$

The optimal level of x given by the condition:

$$B'(x) = C'(x) \tag{4}$$

where B', C' are called the marginal benefit and cost (as derivatives with respect to x). Because $dB/dt = B'(x)\dot{x}$, then it is sufficient to work with (3), as the $\dot{x}$ is a common scaling factor. In Fig A1 we consider the trajectories of the marginal and cumulative costs and benefits under different parameter choices.

In Fig A1(A) we plot the trajectories of the system (1) when $\alpha = 2\alpha_\star$, which is a sustainable regime (a fixed point is reached). In Fig A1(C) we see how the marginal cost and benefit change over time (solved from (3)). At equilibrium, the two quantities are equal at non-zero values. In Fig A1(E), the cost and benefit functions are plotted and their difference tends to a constant, given by the equilibrium value of z.

In Fig A1(B) we plot the trajectories of the system (1) when $\alpha = 2\alpha_c$, which is an unsustainable regime (a limit cycle with large amplitude). In Fig A1(D) we see how the marginal cost and benefit change over time but no longer equalize at non-zero values. In this case, the marginal cost eventually exceeds the marginal benefit until utility (or wealth) decreases to zero. In Fig A1(F), the cost and benefit functions are plotted and we see the system has collapsed once the benefits and costs are equal.

From a mathematical perspective, the above considerations are almost trivial; however, from a psychological perspective, they are not. An economic agent seeking to maximize utility is called "rational". As we can see from the above analysis, a system with $\alpha < \alpha_c$ reaches a stable steady state that maximizes utility with marginal costs equal to marginal benefits. Hence, we can say that the system behaved rationally. However, if $\alpha > \alpha_c$, the system oscillates and collapses (all variables reach close to zero values). In this case, the marginal costs and benefits are no longer equal (at nonzero values). The (cumulative) costs and benefits are equal at the end of a cycle. Hence, the system does not behave rationally, as defined by traditional economic thought.

Passed peak wealth, namely where the marginal cost and benefit cross in Fig A1(D), the system continues to operate until every benefit that was gained in the growth period is lost. This has a historical parallel with the (Western) Roman Empire, which in its later period incurred greater costs (e.g., attacks, revolts) than benefits. The erosion of prior benefits can be observed in the debasement of the denarius, whose silver content was continually reduced. The above analysis provides a contrast between the usual conception of economic rationality, which is sensitive to marginal quantities, and the type of behavior that is more consistent with the observational record of societies that have collapsed. The latter type of behavior is sensitive to cumulative amounts of benefits and costs, akin to what

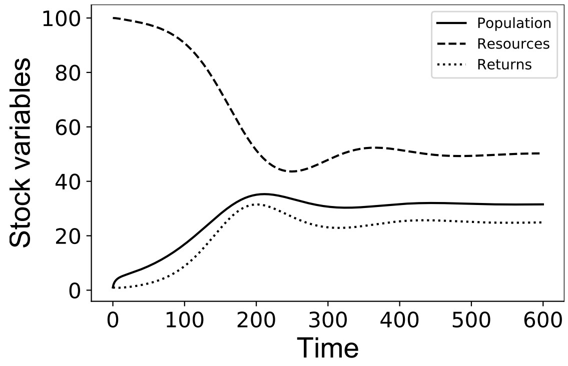

(A)

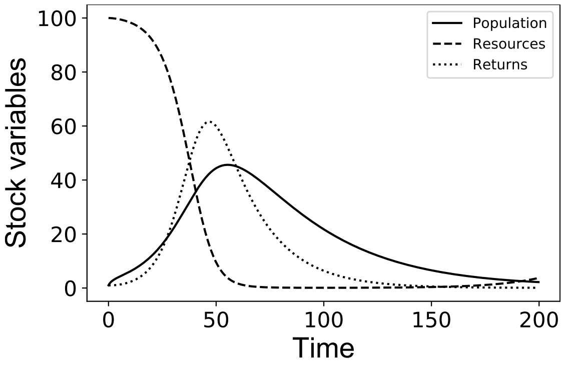

(B)

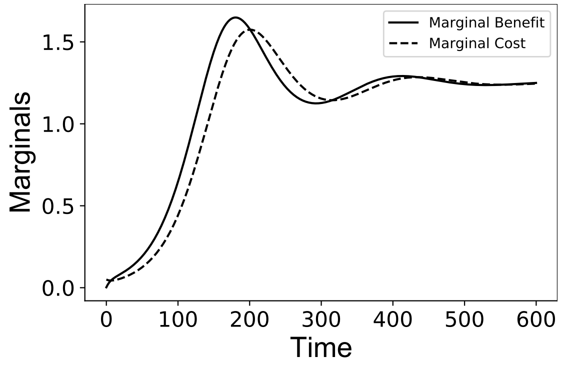

(C)

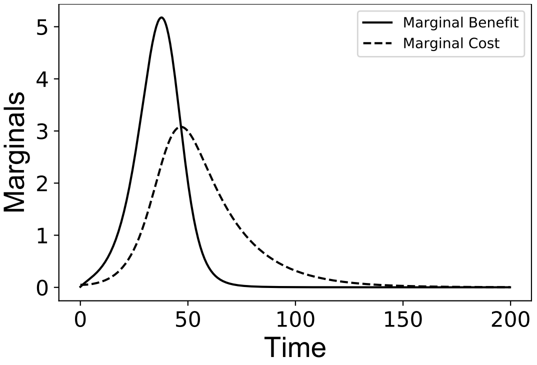

(D)

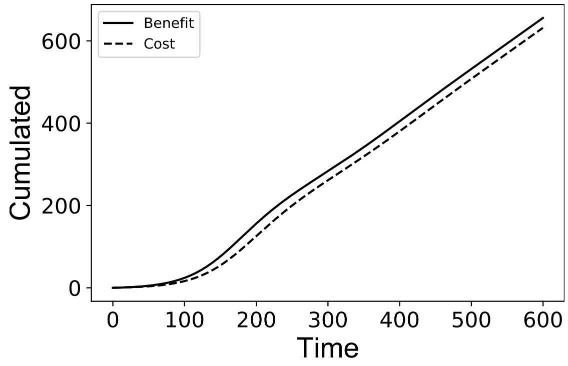

(E)

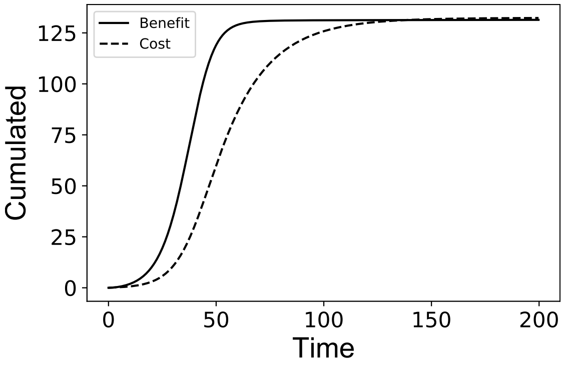

(F)

Figure A1: (Left) Trajectories for a sustainable society (stable fixed point attractor) with $\alpha = \alpha_c$: (A) Population, resources and returns reach fixed points, (C) Marginals costs and benefits are equal at equilibrium, (E) Overall benefits always exceed the costs. (Right) Trajectories for a unsustainable society (large amplitude limit cycle attractor) with $\alpha = \alpha_c$: (B) Population, resources and returns oscillate and reach close to zero values (D) Marginals costs and benefits cross at non-zero values but do not settle there, (F) System evolves till costs equal benefits (all social surplus is gone).

we would expect for sunk-cost effects [1]. The total incurred benefits can be considered sunk costs, and the trajectory of the unsustainable system continues until the real costs equal or exceed the sunk costs.

References

[1] Janssen MA, Kohler TA, Scheffer M. Sunk-Cost Effects and Vulnerability to Collapse in Ancient Societies. Current Anthropology. 2003;44(5).
