# Supplementary material for: Global history, the emergence of chaos and inducing sustainability in networks of socio-ecological systems
Source: PLoS One. 2023 Nov 16;18(11):e0293391. doi: 10.1371/journal.pone.0293391 (PMC10653456; doi:10.1371/journal.pone.0293391)
Supplement: Attachment — Submitted filename: Response_to_reviewers__Plos_One_.pdf [file pone.0293391.s002.pdf]

Response to reviewers

Reviewer 1

The manuscript presents a new dynamical system to model socio-environmental systems with three state variables representing population, resources and wealth. The system is based on a previous model (Easter Island model), but is simpler. A bifurcation diagram based on the extraction rate of resources leading from steady state to limit cycle via Hopf bifurcation and to chaos is presented; a network of coupled oscillators is defined and studied based on a diffusion parameter and the topology. The results delimit parameter ranges where the system becomes sustainable, i.e., the population reaches a nonzero steady state. The oscillatory behavior is to be avoided, since it shows minimum values close to zero, where the system collapses. Overall, the paper is cleary written and the results seem to be correct. I have a few corrections that I would like to see addressed before the paper can be accepted.

The reviewer has provided an accurate synopsis of the paper. We thank them for the close attention they had in reviewing the paper.

1) It is not clear to me if the authors consider the new system as more realistic or not in comparison to Easter Island. Please, comment on that.

The new system we explore in the paper shows enhanced results compared to the Easter Island model. For the Easter Island model, if two systems are coupled together then there is a region of parameter space when one society can be sustainable (reaches a steady state) and another is unsustainable (has a limit cycle) and the overall system can be stabilized. However, the region of parameter space where this occurs is more restricted than for the new model (two coupled systems always seem to stabilize if one of the systems is sustainable). Furthermore, if we take $\alpha_\star$ in each model as a basis for comparison and give the new system a similar parametrization to the Easter Island model (to the extent the parameters can be matched), then in the new model the region of sustainability (reaching a stable fixed point) in the parameter space is much larger by comparison and hence the result is more robust.

Furthermore, the model of Easter Island is specific to that case study, in terms of structure and parametrization. The new system has a more general structure, which keeps some essential features of the Easter Island model but

can have wider applicability (e.g., the x^2 is generally indicative of cooperative behaviour).

We now mention the improvements over the Easter Island model in the Conclusion, 4th paragraph.

2) The terms in the model equation (Equation 1) are well explained, but I did not understand the meaning of the term $-e^{-z/\epsilon}$ in the equation for dx/dt.

We noticed that the term $1 - e^{-z/\epsilon}$ provides a regularization to this system when $z \approx 0$. It ensure that whenever $z \to 0$ it does so more smoothly and does not destabilize the numerical integration method. Without this term the networked dynamical systems are more stiff and more prone to numerical instabilities.

We now mentions these facts in the second paragraph after introducing the system of equations, page 4.

3) In the paragraph right after Eq. (1), "The decrease in resources leads to an increase in returns z". Since alpha¿0, based on the equation for dz/dt I think it should be "The increase in resources leads to an increase in returns z".

The extraction of resources leads to an increase in z. The extraction of resources decreases their amount.

We have rephrased the sentence and the one prior to improve their clarity, first paragraph page 4.

4) In Fig. 1(d), it would help the reading to indicate the position of $\alpha_\star$ and $2\alpha_\star$ in the bifurcation diagram. It becomes clear in the text, but a visual mark would make it easier for the reader.

We have modified Fig. 1(d) to provide a visual cue for the position of $\alpha_\star$ and $2\alpha_\star$.

5) In the caption of Fig. 1, it should be $alpha = 2alpha_\star$, instead of $\alpha = 2a_\star$.

This was a typo. We have corrected it.

6) In table 1, how were the typical values chosen?

The parameters d, r, c where chosen such that the characteristic timescale for decay/regeneration is 20 years (e.g., consistent with swidden agriculture). The parameter $K = 100$ was chosen for numerical convenience. The values of α and b were chosen such that the product αb gives $y_{eq} = \frac{cd}{\alpha b} = K/2$.

7) On page 6, A is defined as a matrix with entries equal to $1/N$, but in the mathematical definition, A=$1_N 1_N^T/N$. I am confused with the notation. Assuming that 1_N is an NxN matrix with ones in all positions, then $1_N * 1_N^T = 5_N$,

so $A = 1$ in all positions. For me, it should be $A = 1_N/N$.

1_N is taken as the N-length column vector of 1s. So $1_N 1_N^T$ gives a $N \times N$ matrix of 1s. We have clarified this in the text, last paragraph page 6.

8) On page 6, second paragraph, "We see the distance has a periodic behavior up to a point and then becomes chaotic". Since chaos has not been demonstrated up to this point in the text, I suggest "We see that the distance has a periodic behavior up to a point and then becomes aperiodic".

We have made the modification, first paragraph page 8.

9) Based on Fig. 2d, the computation of the maximum Lyapunov exponent seems to be based on a single pair of initial conditions, and a short time series. The Lyapunov exponent is an asymptotic result of ergodic systems, so the time series should be long. Since the nearby trajectories will inevitably diverge from the close vicinity of each other in chaotic systems, people usually periodically reorthogonalize the trajectories and rescale their distances in order to keep computing the Lyapunov exponent for a long time (see J. C. Sprott https://sprott.physics.wisc.edu/chaos/lyapexp.htm). Alternatively, the average result from an ensamble of initial conditions should be computed.

We agree with the reviewer. There are two clarifications we need to make. Firstly, the Y-axis in Fig. 2(d) (now 2(f)) is the log of the distance (it covers over 10 orders of magnitude) but this was not indicated. We have corrected this and now show the Y-axis on a logarithmic scale. Secondly, the original result was over an ensemble of initial conditions but we did not quote the uncertainty. We redid the analysis and got an average of $3.4(2) \times 10^{-3}$. However, the greatest source of uncertainty does not come from the initial conditions but rather the choice of interval over which to make the fit. The exponentially divergent behaviour of the trajectories does not necessarily occur immediately from any initial condition, but depends on the location in phase space.

In addition, the exact estimate for our parametrization is less relevant than the range of values the exponent takes over different parametrisations, which gives timescales in the range 300-1000 years.

We have made the necessary changes in the second paragraph on page 8.

To better highlight the emergence of chaos, we have also included a bifurcation diagram in Fig. 2(c), which shows the emergence of multiple extrema for the x_1 variable. In addition, Fig. 2(d) now shows the power spectrum for the periodic and chaotic regimes of the networked system.

10) Since matrix B determines the network topologies shown in Fig. 3, I would like to see the actual matrices employed for each network. That makes it easier to reproduce the results.

The matrix B is the Laplacian of the undirected network, so the node degrees

are on the diagonal, -1 for each edge connection and otherwise the entries are zero.

We have specified this in the first paragraph after introducing the network model, on page 6.

11) The bibliographic references need to be revised. Some errors are pointed below:
Reference 14 - Publisher's name is omitted. I think it is Oxford University Press.
Reference 24 - Journal information is omitted. It is Nature 497, 51-59.
Reference 28 - Publisher's name is omitted.
Reference 33 - There are invalid characters in the doi code.
Reference 38 - page information is strange.
Reference 59 - volume and page information are missing.
Reference 65 - Publisher's name is omitted.
Reference 67 - Publisher's name is omitted.
Reference 71 - Volume information is missing.

All the information has now been provided in the references.

Reviewer 2

In this work, the authors have proposed a three-dimensional model of socio-environmental dynamics that accounts for population, resources and wealth. The topic is hot. The paper is well structured and written. The obtained results are new and very interesting. So, this work merit to be published.

We thank the reviewer for the positive evaluation of the paper and its contribution.
